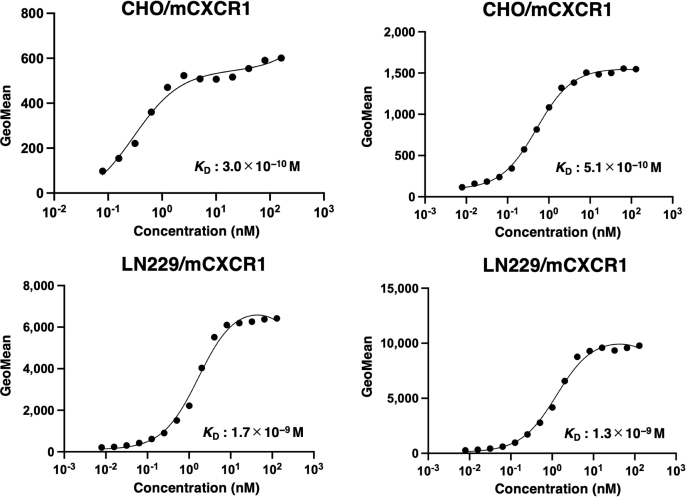# A novel anti-mouse CXCR1 monoclonal antibody, Cx_1_Mab-8, demonstrates nanomolar affinity in flow cytometry

**DOI:** 10.1016/j.bbrep.2025.101965

**Published:** 2025-03-01

**Authors:** Guanjie Li, Hiroyuki Suzuki, Tomohiro Tanaka, Hiroyuki Satofuka, Mika K. Kaneko, Yukinari Kato

**Affiliations:** Department of Antibody Drug Development, Tohoku University Graduate School of Medicine, 2-1 Seiryo-machi, Aoba-ku, Sendai, Miyagi 980-8575, Japan

**Keywords:** Mouse CXCR1, Monoclonal antibody, Peptide immunization, Flow cytometry

## Abstract

CXC chemokine receptor 1 (CXCR1) is an important regulator for neutrophil granulocyte activation through binding to the ligand interleukin-8 (IL-8). Upon binding to IL-8, CXCR1 activates downstream signaling, critical for innate and adaptive immune responses. The IL-8-CXCR1 axis also plays an important role in tumor progression, especially in the tumor microenvironment. CXCR1 antagonists or anti-IL-8 monoclonal antibodies (mAbs) have been developed and evaluated in clinical trials for inflammatory diseases and tumors. In this study, we developed novel mAbs for mouse CXCR1 (mCXCR1) using the N-terminal peptide immunization. Among the established anti-mCXCR1 mAbs, Cx_1_Mab-8 (rat IgG_2b_, kappa) recognized mCXCR1-overexpressed Chinese hamster ovary-K1 (CHO/mCXCR1) and mCXCR1-overexpressed LN229 (LN229/mCXCR1) by flow cytometry. The dissociation constant (*K*_D_) values of Cx_1_Mab-8 for CHO/mCXCR1 and LN229/mCXCR1 were determined as 4.1 × 10^−10^ M and 1.5 × 10^−9^ M, respectively. These results indicated that Cx_1_Mab-8 is useful for detecting mCXCR1 by flow cytometry with high affinity and could contribute to obtaining the proof of concept in preclinical studies.

## Highlights

• A novel anti-mouse CXCR1 monoclonal antibody, Cx_1_Mab-8 were developed by the N-terminal peptide immunization.
• Cx_1_Mab-8 is useful for detecting mCXCR1 by flow cytometry with high affinity.
• Cx_1_Mab-8 could contribute to the preclinical studies for tumor immunotherapy.

## 1 Introduction

CXC chemokine receptor 1 (CXCR1) is a G protein-coupled receptor that plays an essential regulatory role in migration and activation of neutrophil granulocytes [1]. CXCR1 serves as a receptor for interleukin-8 (IL-8, also known as C-X-C motif chemokine ligand 8), a central mediator of immune and inflammatory responses involved in many disorders including cancer [2]. Upon binding to IL-8, CXCR1 triggers a rapid and transient increment of free calcium in neutrophil granulocytes through a GTP-binding protein [2], which results in the migration to the sites of tissue damage or infection [3]. The attracted neutrophil granulocytes kill and phagocytose bacteria at the sites of inflammation. Furthermore, the IL-8-CXCR1 axis plays an essential role in the tumor microenvironment to promote inflammation and resistance to immunotherapy [4]. Therefore, the blockade of the IL-8-CXCR1 axis is a promising strategy to improve antitumor efficacy in combination with other immunotherapies [5].

The activation of CXCR1 involves both N-terminal residues and extracellular loops [3,6]. The structure of human CXCR1 in a lipid bilayer was solved using nuclear magnetic resonance spectroscopy, which facilitated molecular modeling and the understanding of interactions with small molecule inhibitors [7]. The structure of CXCR1 complexed with IL-8 and Gαi1 protein was solved using cryo-EM [8]. The CXCR1 N-terminal residues fit loosely into an IL-8 groove to form the interaction surface chemokine recognition site 1 (CRS1) [8]. Therefore, monoclonal antibodies (mAbs) that recognize the CXCR1 N-terminus are expected to neutralize the IL-8 binding.

We have developed anti-mouse chemokine receptor mAbs against CXCR1 (clone Cx_1_Mab-1) [9], CXCR3 (clone Cx_3_Mab-4) [10], CXCR4 (clone Cx_4_Mab-1) [11], CCR1 (clone C_1_Mab-6) [12], CCR3 (clones C_3_Mab-2, C_3_Mab-3, and C_3_Mab-4) [[13], [14], [15]], CCR5 (clone C_5_Mab-2) [16], CCR8 (clones C_8_Mab-1, C_8_Mab-2, and C_8_Mab-3) [[17], [18], [19]], using the Cell-Based Immunization and Screening (CBIS) method. The CBIS method includes immunizing antigen-overexpressed cells and high-throughput hybridoma screening using flow cytometry. Furthermore, we established anti-murine chemokine receptor mAbs against CCR2 (clone C_2_Mab-6) [20], CCR3 (clones C_3_Mab-6 and C_3_Mab-7) [21], CCR4 (clone C_4_Mab-1) [22], CCR5 (clones C_5_Mab-4 and C_5_Mab-8) [23], CCR9 (clone C_9_Mab-24) [24], CXCR6 (clone Cx_6_Mab-1) [25], and ACKR4 (clones A_4_Mab-1, A_4_Mab-2, and A_4_Mab-3) [26] using the N-terminal peptide immunization.

In this study, a high-affinity anti-mouse CXCR1 (mCXCR1) mAb was developed by N-terminal peptide immunization.

## 2 Materials and methods

### 2.1 Cell lines and plasmids

LN229, Chinese hamster ovary (CHO)–K1, and P3X63Ag8U.1 (P3U1) cell lines were sourced from the American Type Culture Collection (ATCC, Manassas, VA).

A pCMV6neo-myc-DDK plasmid carrying mCXCR1 (Accession No.: NM_178241) was obtained from OriGene Technologies, Inc. (Rockville, MD).

CHO–K1, mCXCR1-overexpressed CHO–K1 (CHO/mCXCR1), and P3U1 cells were maintained in RPMI-1640 medium (Nacalai Tesque, Inc., Kyoto, Japan). LN229 and mCXCR1-overexpressed LN229 (LN229/mCXCR1) were cultured in DMEM (Nacalai Tesque, Inc.). The media were supplemented with 10% FBS, 100 U/mL penicillin, 100 μg/mL streptomycin, and 0.25 μg/mL amphotericin B (Nacalai Tesque, Inc.). All cell cultures were maintained at 37 °C in a humidified incubator with 5% CO_2_ and 95% air.

### 2.2 Peptides

A partial sequence of the N-terminal extracellular domain of mCXCR1 (_1_-MAEAEYFIWTNPEGDFEKE-_19_) with an additional C-terminal cysteine was synthesized by Eurofins Genomics KK (Tokyo, Japan). The peptide was conjugated to keyhole limpet hemocyanin (KLH) at its C-terminus.

### 2.3 Production of hybridomas

A five-week-old Sprague–Dawley rat was sourced from CLEA Japan (Tokyo, Japan). The experimental procedures received approval from the Animal Care and Use Committee of Tohoku University (Permit No.: 2022MdA-001) and adhered to the NIH (National Research Council) Guide for the Care and Use of Laboratory Animals.

The rat was immunized intraperitoneally with 100 μg of KLH-conjugated mCXCR1 peptide (mCXCR1-KLH) combined with 2% Alhydrogel adjuvant (InvivoGen). The immunization regimen consisted of three additional weekly doses (100 μg per rat) and a final booster dose (100 μg per rat) administered two days before the collection of spleen cells. Spleen cells were harvested and fused with P3U1 cells using PEG1500 (Roche Diagnostics, Indianapolis, IN). The supernatants were screened using enzyme-linked immunosorbent assay (ELISA) with the mCXCR1 peptide, followed by flow cytometry analysis with CHO/mCXCR1 and CHO–K1 cells.

### 2.4 Antibodies

Alexa Fluor 488-conjugated anti-rat IgG and peroxidase-conjugated anti-rat IgG were obtained from Cell Signaling Technology, Inc. (Danvers, MA) and Sigma-Aldrich Corp. (St. Louis, MO), respectively.

### 2.5 ELISA

The mCXCR1 peptide (MAEAEYFIWTNPEGDFEKEC) was immobilized onto Nunc Maxisorp 96-well plates (Thermo Fisher Scientific Inc.). The wells were blocked with PBS containing 0.05% Tween 20 and 1% bovine serum albumin (BSA). Following this, the plates were incubated with supernatants from hybridoma cultures, followed by peroxidase-conjugated anti-rat IgG at a dilution of 1:20,000. The enzymatic reactions were subsequently carried out using the ELISA POD Substrate TMB Kit (Nacalai Tesque, Inc.).

### 2.6 Flow cytometric analysis

Cells were collected following brief treatment with 0.25% trypsin and 1 mM ethylenediaminetetraacetic acid (EDTA; Nacalai Tesque, Inc.). Afterward, the cells were rinsed with a blocking buffer of 0.1% BSA in PBS and incubated with varying concentrations (0.01, 0.1, 1, and 10 μg/mL) of Cx_1_Mab-8 for 30 min at 4 °C. Subsequently, the cells were exposed to Alexa Fluor 488-conjugated anti-rat IgG diluted to 1:2,000. Fluorescence measurements were then obtained using the SA3800 Cell Analyzer (Sony Corp.).

### 2.7 Determination of dissociation constant (*K*_D_) by flow cytometry

CHO/mCXCR1 cells were incubated in a series of diluted solutions of Cx_1_Mab-8 for 30 min at a temperature of 4 °C. Following this, the cells were treated with Alexa Fluor 488-conjugated anti-rat IgG at a dilution of 1:200. Fluorescence measurements were then obtained using the SA3800 Cell Analyzer. The *K*_D_ was determined by GraphPad PRISM 6 software (GraphPad Software, Inc., La Jolla, CA).

## 3 Results

### 3.1 Development of Anti-mCXCR1 mAbs using the immunization of N-terminal peptide

**Fig. 1:**
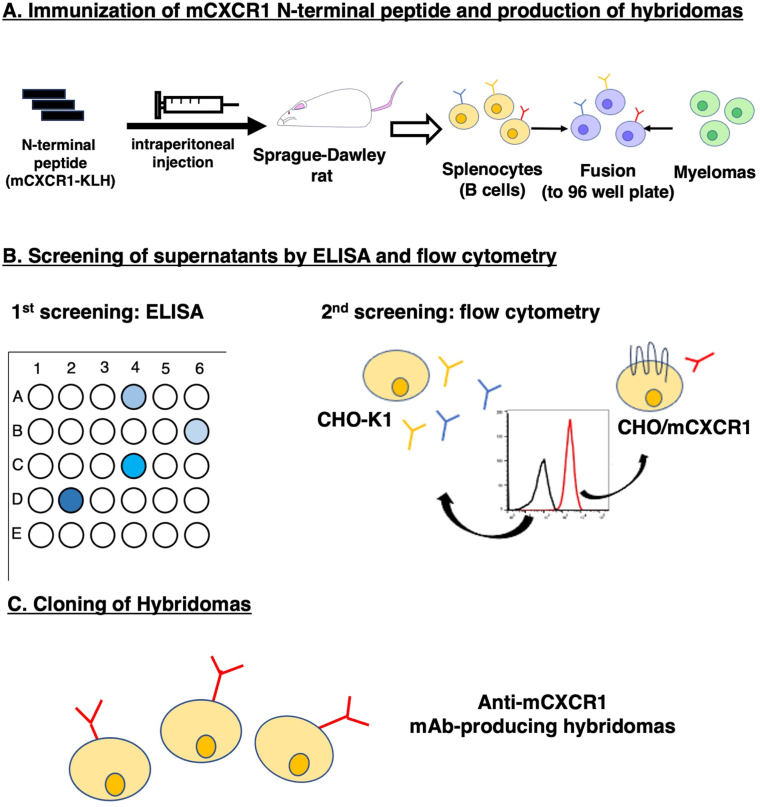
A schematic procedure of anti-mCXCR1 mAbs production. (**A**) mCXCR1 N-terminal peptide conjugated with KLH (mCXCR1-KLH) was immunized into a Sprague–Dawley rat. The spleen cells were fused with P3U1 cells. (**B**) To select anti-mCXCR1 mAb-producing hybridomas, the supernatants were screened by ELISA and flow cytometry using CHO–K1 and CHO/mCXCR1 cells. (**C**) After limiting dilution, an anti-mCXCR1 mAb, Cx_1_Mab-8, was finally established. ELISA, enzyme-linked immunosorbent assay.

To generate anti-mCXCR1 mAbs, a rat was immunized with mCXCR1-KLH (Fig. 1A). Following immunization, the spleen was excised from the rat, and the splenocytes were fused with myeloma P3U1 cells. The resulting hybridomas were then plated into ten 96-well plates and cultured for six days. Subsequently, positive wells to the mCXCR1 peptide were identified using ELISA, followed by the selection of supernatants that were reactive to CHO/mCXCR1 and non-reactive to CHO–K1 via flow cytometry (Fig. 1B). The ELISA screening revealed that 102 out of 958 wells (10.6%) exhibited a strong reaction with the mCXCR1 peptide. Flow cytometric analysis further identified 8 out of these 102 wells (7.8%) that demonstrated strong signals with CHO/mCXCR1 cells but not with CHO–K1 cells. After conducting limiting dilution and several additional screenings, Cx_1_Mab-8 (rat IgG_2b_, kappa) was successfully established (Fig. 1C).

### 3.2 Flow cytometry using Cx_1_Mab-8

**Fig. 2:**
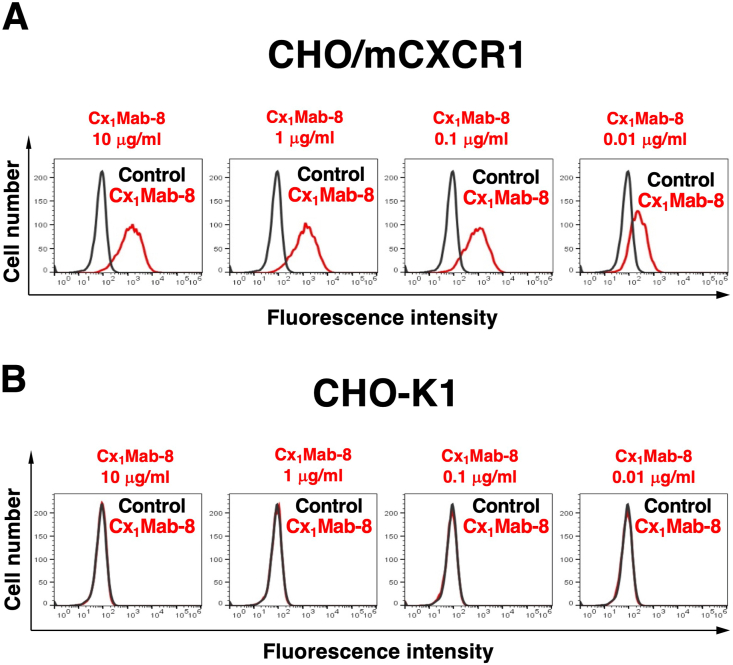
Flow cytometric analysis of Cx_1_Mab-8 against CHO/mCXCR1 and CHO–K1. CHO/mCXCR1 (A) and CHO–K1 cells (B) were treated with 0.01–10 μg/mL of Cx_1_Mab-8, followed by Alexa Fluor 488-conjugated anti-rat IgG. Fluorescence data were subsequently collected using the SA3800 Cell Analyzer.

**Fig. 3:**
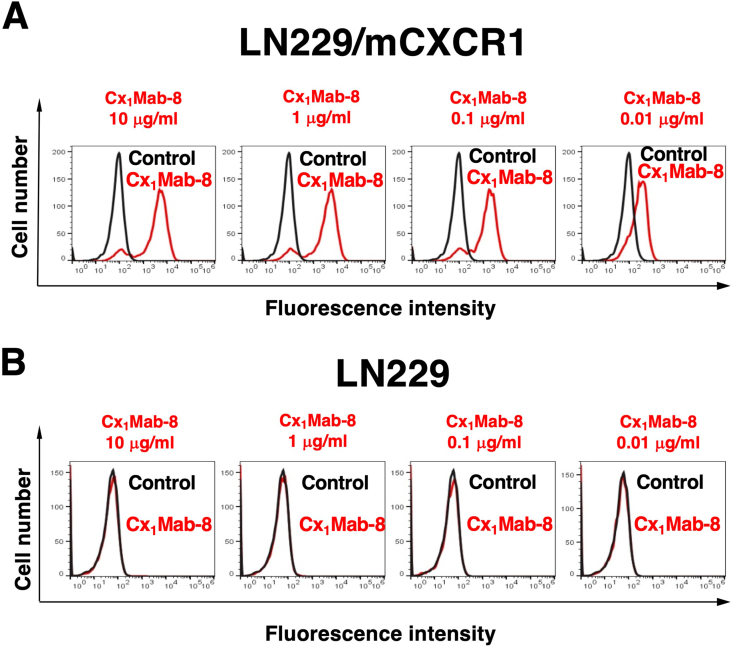
Flow cytometric analysis of Cx_1_Mab-8 against LN229/mCXCR1 and LN229. LN229/mCXCR1 (A) and LN229 cells (B) were treated with 0.01–10 μg/mL of Cx_1_Mab-8, followed by Alexa Fluor 488-conjugated anti-rat IgG. Fluorescence data were subsequently collected using the SA3800 Cell Analyzer.

We conducted flow cytometry using Cx_1_Mab-8 against CHO/mCXCR1 and CHO–K1 cells. Cx_1_Mab-8 dose-dependently recognized CHO/mCXCR1 cells at 10, 1, 0.1, and 0.01 μg/mL (Fig. 2A). Cx_1_Mab-8 did not recognize parental CHO–K1 cells even at 10 μg/mL (Fig. 2B). The similar reactivity of Cx_1_Mab-8 was also observed in LN229/mCXCR1 cells (Fig. 3).

### 3.3 The binding affinity of Cx_1_Mab-8

**Fig. 4:**
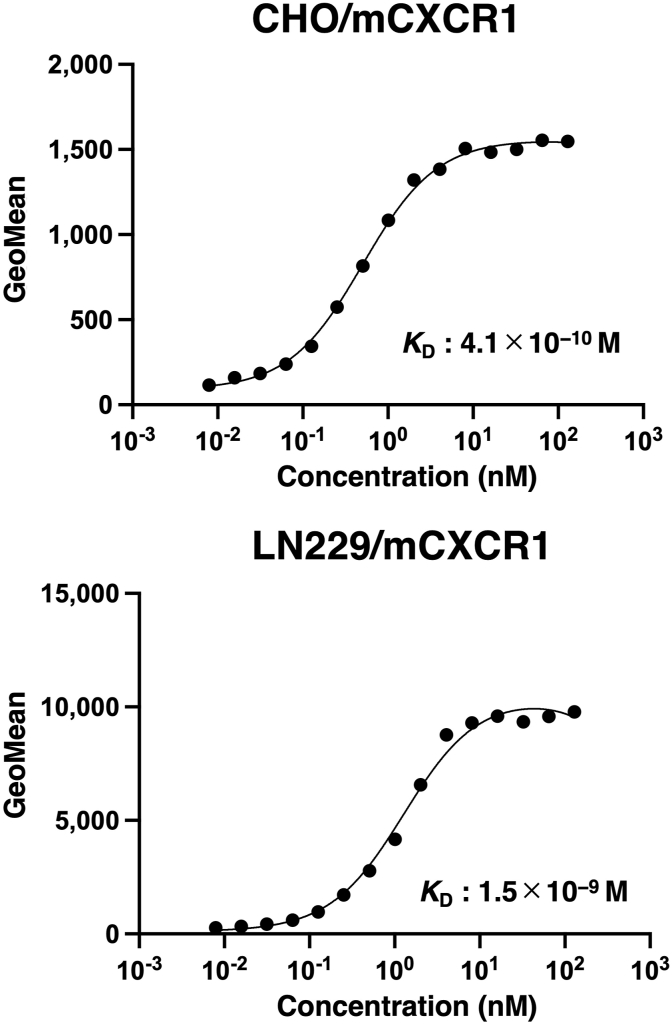
The determination of the binding affinity of Cx_1_Mab-8. CHO/mCXCR1 (A) or LN229/mCXCR1 (B) cells were suspended in 100 μL serially diluted Cx_1_Mab-8. Then, cells were treated with Alexa Fluor 488-conjugated anti-rat IgG. Fluorescence data were subsequently collected using a SA3800 Cell Analyzer, following the calculation of the dissociation constant (*K*_D_) by GraphPad PRISM 6. The average *K*_D_ values of Cx_1_Mab-8 for CHO/mCXCR1 and LN229/mCXCR1 were determined from two independent measurements.

We conducted flow cytometry to determine the *K*_D_ values of Cx_1_Mab-8 against CHO/mCXCR1 and LN229/mCXCR1. The average *K*_D_ values of Cx_1_Mab-8 for CHO/mCXCR1 and LN229/mCXCR1 from two independent measurements (Fig. S1) were determined as 4.1 × 10^−10^ M and 1.5 × 10^−9^ M, respectively (Fig. 4).

**Fig. 5:**
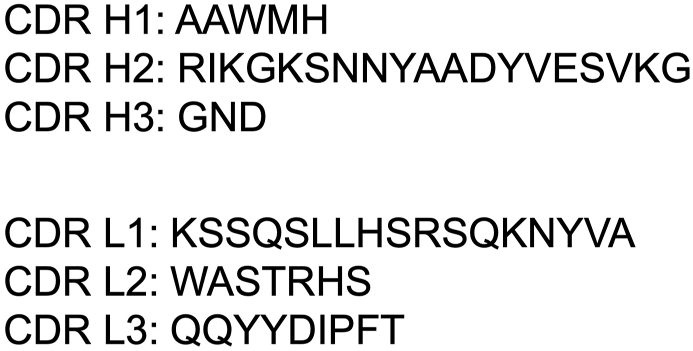
Amino acid sequence of complementarity-determining regions of Cx_1_Mab-8. The cDNA of Cx_1_Mab-8 variable regions was cloned, and the amino acid sequences of complementarity-determining regions were determined.

We cloned the cDNA of Cx_1_Mab-8 variable regions and showed amino acid sequences of complementarity-determining regions (Fig. 5).

## 4 Discussion

In this study, we developed a novel anti-mCXCR1 mAb, Cx_1_Mab-8, using the N-terminal peptide immunization and showed the usefulness of flow cytometry (Fig. 2, Fig. 3) to detect mCXCR1. Cx_1_Mab-8 possess superior affinities: 4.1 × 10^−10^ M (CHO/mCXCR1) and 1.5 × 10^−9^ M (LN229/mCXCR1), respectively (Fig. 4) compared to that of a previously established anti-mCXCR1 mAb, Cx_1_Mab-1: 2.6 × 10^−9^ M (CHO/mCXCR1) and 2.1 × 10^−8^ M (LN229/mCXCR1) [9].

As described in the result section, less than 10% of ELISA-positive supernatants recognized CHO/mCXCR1 in flow cytometry. One possible explanation is a disulfide bond connecting the CXCR1 N-terminus (Cys30 in humans) to the extracellular start of transmembrane 7 (Cys277) [7,8]. The Cys pair is highly conserved in the chemokine receptors and is essential for ligand binding. Furthermore, it plays a critical role in shaping the extracellular structure of the chemokine receptors and provides a restriction for the structure formation. Therefore, determining the Cx_1_Mab-8 epitope is essential to understand the recognition of mCXCR1. We previously identified the Cx_6_Mab-1 epitope using 1 × and 2 × alanine scanning methods [27]. Future studies should focus on determining the epitope of Cx_1_Mab-8.

The N-terminus of chemokine receptors plays an essential role in chemokine specificity. Structural studies have shown that the receptor N-terminus binds to the chemokine core at an interface of CRS1. In contrast, the chemokine N-terminus fits within a pocket of the receptor's TM helical domain (called CRS2) [28,29]. HuMax-IL8 (BMS-986253) is a fully human monoclonal antibody against IL-8. HuMax-IL8 inhibits tumor progression by suppressing IL-8-mediated epithelial-mesenchymal transition, immune escape, and recruitment of myeloid-derived suppressor cells [30]. A clinical trial is currently underway in hormone-sensitive prostate cancer, examining its combination with nivolumab in patients with rising prostate-specific antigen [31]. Although CXCR1 antagonists such as navarixin and reparixin have been developed for asthma, pneumonia, and solid tumors [5], mAb therapy using anti-CXCR1 has not been explored. Further studies are needed to investigate the neutralizing activity of Cx_1_Mab-8 against murine IL-8 orthologues, including KC, MIP-2, and LIX [32]. Since we successfully identified the complementarity-determining regions of Cx_1_Mab-8 (Fig. 5), class-switched mAbs of Cx_1_Mab-8 to mouse immunoglobulins could facilitate preclinical studies for inhibiting mCXCR1 or depletion of mCXCR1-positive cells in mouse models.

## CRediT authorship contribution statement

**Guanjie Li:** Investigation. **Hiroyuki Suzuki:** Writing – original draft, Investigation, Funding acquisition. **Tomohiro Tanaka:** Investigation, Funding acquisition. **Hiroyuki Satofuka:** Investigation, Funding acquisition. **Mika K. Kaneko:** Investigation. **Yukinari Kato:** Writing – review & editing, Project administration, Funding acquisition, Conceptualization.

## Author disclosure statement

The authors have no conflict of interest.

## Funding information

This research was supported in part by 10.13039/100009619Japan Agency for Medical Research and Development (10.13039/100009619AMED) under Grant Numbers: JP24am0521010 (to Y.K.), JP24ama121008 (to Y.K.), JP24ama221339 (to Y.K.), JP24bm1123027 (to Y.K.), and JP24ck0106730 (to Y.K.), and by the 10.13039/501100001691Japan Society for the Promotion of Science (10.13039/501100001691JSPS) Grants-in-Aid for Scientific Research (10.13039/501100001691KAKENHI) grant nos. 22K06995 (to H.Suzuki), 24K18268 (to T.T.), 24K11652 (to H.Satofuka), and 22K07224 (to Y.K.).

## Declaration of competing interest

The authors declare the following financial interests/personal relationships which may be considered as potential competing interests:Yukinari Kato reports financial support was provided by 10.13039/100009619Japan Agency for Medical Research and Development. Hiroyuki Suzuki reports financial support was provided by 10.13039/501100001691Japan Society for the Promotion of Science. Tomohiro Tanaka reports financial support was provided by 10.13039/501100001691Japan Society for the Promotion of Science. If there are other authors, they declare that they have no known competing financial interests or personal relationships that could have appeared to influence the work reported in this paper.

## Figures and Tables

**figs1:**